# Commentary: Protective effect of quercetin on kidney diseases: from chemistry to herbal medicines

**DOI:** 10.3389/fphar.2023.1214664

**Published:** 2023-05-30

**Authors:** Weiya Kong, Yage Zhang, Liangchen Luo, Qiaoyin Tan

**Affiliations:** ^1^ School of Sports Medicine and Rehabilitation, Beijing Sport University, Beijing, China; ^2^ Fifth Affiliated Hospital of Harbin Medical University, Daqing, China; ^3^ College of Teacher Education, Zhejiang Normal University, Jinhua, Zhejiang, China

**Keywords:** quercetin, bioflavonoids, natural product, kidney injury, renal disease, kidney diseases, healthcare

## 1 Introduction

Kidney disease is a life-threatening condition with a high mortality rate that can be caused by various factors, such as nephrotoxins, oxidative stress, or inflammation. These factors may be the main causes that promote renal injury towards fibrosis, ultimately leading to chronic kidney disease (CKD) or end-stage renal disease (ESRD) (Levey and Coresh, 2012). However, the drugs and treatment strategies currently available for treating kidney injury are still very limited.

Quercetin is a natural flavonoid with antioxidant, antihypertensive, and antidiabetic effects. Quercetin is believed to have beneficial effects in the treatment of cancer, cardiovascular disease, and metabolic disorders (Rakha et al., 2022).

We recently read a review article by Chen et al. This comprehensive review paper on the protective effects of quercetin against kidney disease covers many aspects, from pharmacokinetics and bioavailability of quercetin in traditional herbal medicine to the protective effects of quercetin against different types of kidney disease, such as nephrotoxicity, acute and chronic kidney injury, diabetic nephropathy, renal aging, and other rare kidney diseases. Overall, this is a systematic and detailed review aimed at showcasing the potential value of quercetin in the treatment of kidney disease.

## 2 Limitations

The most significant flaw of this study is that it summarizes multiple academic research results in a fragmented way, and the selected relevant studies are limited, and does not form a clear argument. Firstly, the article focuses too much on listing results when analyzing different research findings, neglecting the discussion of the mechanisms themselves. For example, when discussing the relationship between chemical structure and biological activity, the corresponding relationship is not explained clearly and systematically enough. At the same time, the discussion of renal fibrosis is more focused on the inhibition of transforming growth factor β (TGF-β), but other mechanisms and factors that promote renal fibrosis are not further discussed. Do these factors interact with quercetin? Secondly, there are limitations in selecting relevant studies in the article. For example, in the section on diabetic nephropathy, further clarifications on GluT4 and AMPK signaling and EFK-related mechanisms in recent years can be introduced for hypoglycemic effects; for antioxidant effects, the interaction between the peroxisome pathway and Nrf2 and other important antioxidant pathways in diabetic nephropathy (DN) and quercetin can be introduced; for autophagy promotion, the changes in autophagy-related LC3 and the expression of other related proteins can be systematically discussed. In addition, there is a lack of analysis and comparison of different research results and opinions throughout the article, to form a more balanced and thorough conclusion.

Another drawback that has been highlighted is that the article downplays the fact that quercetin cannot yet be used for the clinical prevention and treatment of kidney disease, which may mislead clinical decision-making. Firstly, the review is too biased towards affirming the efficacy of quercetin, without fully demonstrating its toxicological characteristics and safe dosage range, and potential adverse reactions or risks. Even if it is very promising, reliable pharmaceutical pathways are yet to be found, making it difficult to achieve safety and efficiency. Secondly, most of the evidence in the review comes from animal experiments, and the judgment on human applicability is still inadequate. Appropriate clinical studies are still needed, and the conclusion appears to be too hasty. Thirdly, despite the excellent mechanistic evidence, it is still difficult to derive reasonable medication indications and differentiated treatment strategies in practical applications. The lack of in-depth analysis of the interaction between different factors such as different races, types of diseases, and disease courses largely limits its practical application.

## 3 Discussion

Although this review presents a highly promising treatment concept, it still needs to overcome several significant obstacles before it can be recommended as standard treatment. I hope that further research and rigorous testing can overcome its shortcomings and make the conclusion more reliable and cautious. Overall, this is a groundbreaking study, but appropriate caution needs to be taken at different stages to maximize its potential while minimizing unnecessary risks. In this process, further research and testing will be indispensable, and we look forward to it together.
